# ACCESS climate data management

**DOI:** 10.1007/s13280-017-0963-1

**Published:** 2017-10-24

**Authors:** Øystein Godøy, Bard Saadatnejad

**Affiliations:** Norwegian Meteorological Institute, P.O. Box 43, Blinderen, 0313 Oslo, Norway

**Keywords:** Data, Interoperability, Management, Preservation

## Abstract

Building on achievements and experience gained through the EU project DAMOCLES and international data management during the International Polar Year, ACCESS, data management was implemented using the same platform as used for DAMOCLES. A metadata-driven approach through which all datasets are properly described with discovery and use metadata was chosen in order to simplify data management and data usage. The system provides automated submission and checking of datasets, search and download as well as visualisation and transformation on user demand and metadata export. Long-term management of ACCESS climate datasets is done within the context of the Arctic Data Centre. This ensures visibility of ACCESS datasets in the context of WMO and GEOSS catalogues. Challenges with ACCESS data management have mainly been cultural with the consequence that the system has been underutilised within the duration of the project duration.

**Electronic supplementary material:**

The online version of this article (doi:10.1007/s13280-017-0963-1) contains supplementary material, which is available to authorized users.

## INTRODUCTION

Data management is crucial in science. This is irrespective of whether management is done in a structured and well designed manner or on a more ad hoc basis. It covers management of the information collected and generated by science. What was observed, by whom and when? What were the conditions under which the information was gathered and how was the information processed for further use? All scientists do some form of data management, but in recent years, especially with the emerging strength of computer systems and networks, data management has taken a more structured approach in science. Much has been learned from the library and archive communities, but still there are many challenges remaining.

Data-intensive science is a rather new and emerging field. It is sometimes described as the “4th paradigm” of science (Bell et al. 2009), adding to theoretical, empirical and computational science on the science workbench. Following the ideas outlined by Gray (2009), the 4th paradigm of science is data exploration. It is about exploring and exploiting old and new data. Advances in modern computer systems (processing and storage) coupled with development of new software tools have simplified the combination and analysis of datasets. Gray (2009) defines this as e-Science—“where IT meet scientists”.

Following this, the main questions within Earth Sciences are (1) whether there are obstacles preventing data exploration; and (2) how can data exploration be improved? These questions are discussed in the context of experiences gained working with data management in two EU Framework (FP) 6 and FP7 projects, Developing Arctic Modelling and Observing Capabilities for Long-term Environmental Studies (DAMOCLES) and Arctic Climate Change, Economy and Society (ACCESS) related to the question above. Within these projects a number of technical and cultural challenges were identified and the intention of this paper is to raise the awareness of these challenges among both data managers and scientists. The main purpose of DAMOCLES was to establish an integrated ice-atmosphere–ocean monitoring and forecasting system. The main objective was to observe, understand and quantify climate changes in the Arctic. In ACCESS, the main objective was to assess effects of climate change on marine transportation, fisheries and oil and gas exploitation. The main difference between DAMOCLES and ACCESS was a much stronger emphasis on economy and social sciences in ACCESS and less focus on the observational component.

Within the EU FP6 project DAMOCLES,^1^ a data management system was developed to tie the various parts of the project together. This system was originally planned to only cater for the needs of the project, but as DAMOCLES was approved as an International Polar Year (IPY) project, further development of the data management system was necessary in order to cater for the demands of the network-oriented approach adopted in the IPY data management (Parsons et al. 2011a). When EU FP7 project ACCESS was planned, the data management activities were split into two subsystems. The data management system developed in DAMOCLES was integrated as the sub-system for climate-oriented data, while a parallel system for non-climate data was hosted by NERC (Natural Environment Research Council) in the UK. All data collected in DAMOCLES were included in the ACCESS data portal.

While the data management system presented herein was originally developed to support the EU project DAMOCLES, it has been further developed using internal funding at the Norwegian Meteorological Institute as well as funding from the Research Council of Norway to support the basic requirements identified and addressed, but not resolved in DAMOCLES.

A further development of the system is today used to support the WMO Data Collection and Production Centre—the Arctic Data Centre. Following the end of the ACCESS project, the project-based data management system will live until hardware or software requires updates or replacement. Then the project-specific ACCESS data portal will be removed. Data stewardship will continue within the Arctic Data Centre,^2^ and datasets will be tagged as DAMOCLES or ACCESS datasets to simplify data identification.

## PURPOSE

Scientific data management, when properly implemented and utilised, is a mechanism to share data, to coordinate efforts in order to avoid duplication of efforts (if not explicitly required) and to maximise the return on investment of the public in science. In short, data management provides a scientific memory complementary to the memory established through peer-reviewed papers. Through sharing of data, scientific statements and algorithms may be reviewed, quality controlled and further developed. It opens opportunities for innovation, as new perspectives may be used in the analysis and interpretation of data. This implies moving from a “publish and forget” data life cycle, where the main focus is the scientific paper, to the data exploitation life cycle. In the first life cycle, data are collected and stored locally. References to the scientific work of others are included using literature only. Adding data publishing to the process means that data collected can be reused (and referenced), considerably increasing the benefits of public investment in science (Nosek et al. 2015; Wilkinson et al. 2016; Pasquetto et al. 2017).

The purpose of the data management system established for DAMOCLES, and continued in ACCESS, was to establish a project memory, to ensure data sharing within the project and externally with other science communities (whether these communities were considered relevant or not by the internal scientific community) and finally to ensure the long-term data preservation. Initially the main focus was on internal data sharing, but as DAMOCLES also became an endorsed IPY project, the scope was extended to sharing beyond the project and long-term data preservation was emphasised in accordance with IPY requirements^3^ (IPY Data Policy 2008).

The requirement to share data is not new. Prior to digital data becoming the norm, it was common to publish data in tables as part of the papers or reports published. This was normally done in appendices to the papers or reports. However, in the transition into the digital era, this data publishing was more or less forgotten. This was perhaps due the increased volume and complexity of the data. Increasingly, scientific journals (e.g., *Science*) require that data be shared (Nosek et al. 2015). This ensures the ability to quality control the science through reproduction of analyses. Funding agencies are also promoting free and open research data (e.g., European Open Research Data Pilot^4^ and FAIR principles). This is based on an intention to increase the scientific production based on the public funding of science. A prerequisite for achieving the ability to effectively share and reuse data is interoperability at metadata and data levels (Wilkinson et al. 2016). This is related not only to technical, but also cultural issues. Some of these are discussed below.

DAMOCLES and ACCESS data management were initially not focused on distributed data management, i.e., combination of data from physically separated data centres. Neither was the data management in DAMOCLES designed to support distributed data management. However, external regional (e.g., INSPIRE,^5^ SAON/IASC Arctic Data Committee^6^) and global (e.g., WMO Information System,^7^ GEOSS^8^) data management frameworks/initiatives are pushing towards distributed data management (Pulsifer et al. 2014). The system established in DAMOCLES was thus developed into a system supporting distributed data management and has since been used to support a number of distributed data management activities in the context of IPY, WMO (e.g., Global Cryosphere Watch) and national e-infrastructure activities like the Norwegian Satellite Earth Observation Database for Marine and Polar Research (NORMAP), the Norwegian Marine Data Centre (NMDC), the emerging Svalbard Integrated Arctic Earth Observing System (SIOS) and the Norwegian Scientific Data Network. This functionality was also relevant for ACCESS, where the non-climate data were hosted by NERC, but it was never fully exploited for various reasons. While distributed data management removes political issues related to centralising datasets in a limited number of data centres, it introduces challenges related to interoperability between data centres. Whether the centralised or the decentralised approach is most cost efficient is not straightforward to determine as business models for data centres vary. Although interoperability at the datasets level in principle should be easier to achieve in a centralised approach that has full control of the data submission process, this is not always true. Many data centres, for legacy reasons, place greater emphasis on storing the data than actually making them useful in an integrated context (e.g., through combination of various datasets).

## DEVELOPMENT HISTORY AND FUNCTIONALITY

Soon after its launch, DAMOCLES was considered to be a European contribution to the International Polar Year (IPY). IPY data management was coordinated through IPY Data and Information Service (IPYDIS). IPYDIS was a truly distributed data management framework, coordinated through the exchange of discovery metadata following the Global Change Master Directory (GCMD) Directory Interchange Format (DIF) standard. DAMOCLES data were included in the IPY catalogues using the metadata exchange described above. Experiences of IPYDIS data management are documented in Parsons et al. (2011a). IPYDIS did not attain the desired degree of data interoperability, but certainly made progress with metadata interoperability between contributing data centres. Within ACCESS the same system as in DAMOCLES was used, but it was slightly modified and adapted. The two main components of the system were a system for metadata handling, data submission and discovery called METAMOD combined with data access through a THREDDS Data Server.^9^ A THREDDS Data Server is a web server that offers access through various data access protocols to real-time and archived data (UNIDATA 2017a).

The DAMOCLES and ACCESS data management solution was a centralised solution. In ACCESS, there were two data repositories: one for climate data and one for non-climate data. These were not connected. However, as long as scientists did not submit much data to either of the systems, one could argue that there was no need for this. No formal analysis of the lacking support of the ACCESS scientific community was done, but the issue of user uptake of information systems has been addressed by several studies (Markus and Keil 1994; Finholt and Birnholtz 2006; Ribes and Finholt 2008; Mayernik et al. 2013). A very relevant study in this context was carried out by Cutcher-Gerschenfeld et al. (2016) in relation to the EarthCube project in the US since the user community for EarthCube is quite similar to the user community of DAMOCLES and ACCESS. Their conclusion was that there is a gap between the “perceived importance of data sharing in the geosciences and the perceived ease of doing so” (see their Fig. 1). During DAMOCLES it was experienced that reviewers were using the data management system actively and questioned the lack of availability of data in the data management system if deliverable reports claimed data to be collected and analysed. Such inquiries from reviewers were never received in ACCESS resulting in submission of fewer datasets than planned to the data management system. Apparently, the active usage of the data management system by project reviewers was a strong incentive for scientists to actually deposit data in the data management system. Parsons et al. (2011a) discussed incentives for data sharing from the perspective of experiences gained during the International Polar Year and indicated that a combination of “carrots and sticks” is required. Citation of datasets in combination with strict requirements from funding agencies was emphasised as crucial to the promotion of data sharing.

**Fig. 1:**
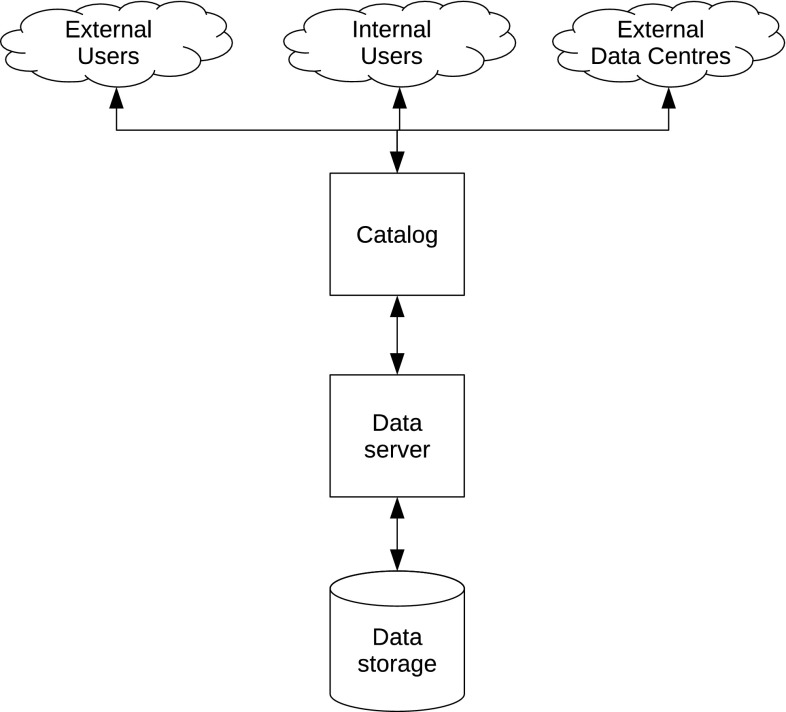
System outline for the data management system established in DAMOCLES and ACCESS

The main functionalities implemented in DAMOCLES and ACCESS data management were data submission, data discovery and data access. Analysing recent trends in scientific and operational data management identified the requirement that the system support distributed data management (e.g., this was what happened during IPY). This means that datasets are not necessarily hosted by one physical data centre, but that they may be served by a virtual data centre integrating data from a number of physically separated data centres. Such data centres are connected using internationally accepted documentation standards and interfaces to metadata and data. In the context of this data management system, the ability to relate to other data centres in a physically distributed network, required implementation of mechanisms to generate and exchange standardised discovery metadata. This required modifications of the system’s metadata structures, but since the system already utilised standardised vocabularies to describe parameters, etc., this modification was simplified.

Data submission was supported through a submission service accepting only standardised data. Upon data submission, the data were checked for conformance and a message was returned to the data provider. The file format to use was determined within the EU FP6 project DAMOCLES. Within this project, an evaluation of various file formats was done. The evaluation emphasised the need for standardised data, i.e., not inventing a DAMOCLES-specific file format, but reusing something already existing, and potentially extending it to support new data types. In this process, relevant background information, whether formal (e.g., Brown et al. 1993) or informal through communication with the user community was evaluated. Brown et al. (1993) describe in their introduction some of the challenges with traditional methods for handling scientific data. The issues they raise are even more relevant today, especially with the distributed data management emerging. Acknowledging that the bulk of data was in the environmental domain, and more so in the climate domain, an obvious candidate was the Network Common Data Format (NetCDF) file format (e.g. Hankin et al. 2009).^10^ However, NetCDF is only a container and essentially no different from ASCII, XML, (Geo)JSON, HDF, etc., in regard to the structured description of its contents. Within the NetCDF community there has been a considerable effort to standardise how the NetCDF container is used for data from the oceanographic and atmospheric domains.^11^ This was first known as the COARDS (named by the sponsor—Cooperative Ocean/Atmosphere Research Data Service) convention, but has since been superseded by the Climate and Forecast (CF) Convention.^12^ Within DAMOCLES, COARDS was evaluated for use first, but the momentum of the CF convention was larger and more future oriented as it also better addressed the more complex nature of data types in the project. The CF convention ensures that data are encoded with sufficient use metadata. Use metadata identifies the variables, the units of a variable, how missing values are encoded, etc. It also addresses the issue of semantics through the application of a controlled vocabulary for description of variables, the CF standard names. Use metadata are required to fully understand and use the data found. It is a standardised description of the content (variables, units, missing values encoding, etc.). In essence, datasets encoded according to the CF convention are self-describing allowing computers to read and understand the data. Furthermore, NetCDF is related to UNIDATA’s Common Data Model^13^ which is also used by the OPeNDAP protocol. To simplify the work for scientists providing data, a file compliance checker was added to the service. This way, the user could upload test files and get an evaluation of these before submitting the full dataset, the ingesting of which could potentially fail. The file compliance checker evaluated conformance with the CF conventions (adapted to CF-1.0) and the global attributes described below. Later, it was discovered that several well-developed file conformance checkers existed and could be used, but these were not easy to fully integrate in the solution developed. Lacking integrated data submission tools complicates the data submission process for data providers as they have to use two different systems: one for checking and one for submission.

The CF conventions does not, however, ensure the presence of discovery metadata. Discovery metadata are used to build catalogues that simplify the data discovery process for data consumers. Within DAMOCLES, the problem of appending discovery metadata to the actual data was solved by defining a number of global attributes (Table S1) to the NetCDF/CF files. These attributes were sufficient to extract metadata compliant with NASA’s GCMD DIF.^14^ The data submission service extracted discovery metadata directly from the datasets submitted and made these available in a searchable catalogue implemented using the PostgreSQL Relational Database Management System. Connected to this database were interfaces for exchange of metadata with external systems using the Open Archives Initiative Protocol for Metadata Harvesting (OAI-PMH^15^). This was, for example, used to connect the DAMOCLES data catalogue to the IPY Data and Information Service (IPYDIS). It proved to be a low-cost and very efficient mechanism for exchanging discovery metadata for datasets and has since been adopted by many data centres contributing to the WMO Information System (WMO 2015). The human interface for searching datasets was based on spatio-temporal search options as well as GCMD science keywords, institutional ownership of data and data features like type of data. The usage of project-specific global attributes^16^ for discovery metadata records within DAMOCLES was not the preferred solution as it was non-standard and mainly developed as a stopgap solution for internal use. When ACCESS started, we found that the NetCDF Attribute Convention for Dataset Discovery (ACDD^17^) was available. The first release of ACDD was available in 2005,^18^ but this was not known to the development team. Adaptations to the software were made in ACCESS allowing users to submit data using CF-1.6 and higher. Furthermore, support for ACDD global attributes was added instead of the DAMOCLES-specific attributes. CF-1.6 and higher is more suitable for observational data as it has standardised models for station time series, profiles and trajectories. All data were made available for external users through a THREDDS Data Server.^19^ During DAMOCLES this was normally set up to serve data using HTTP and OPeNDAP. Aggregation over physical files belonging to the same dataset, for example, in order to establish a virtual file for the whole time series, was not used. In ACCESS, aggregation in time was normally used and OGC Web Map Service was activated for visualisation of gridded products.

The outline of the system developed is provided in Fig. 1. It illustrates the various user communities addressed and how the various components are connected. Users, whether internal or external data consumers, internal data providers or external projects or catalogues, connect to the catalogue through human or machine interfaces serving discovery metadata. These metadata describe the datasets hosted, and provide interfaces to the actual data. Data were served using a dedicated application server (THREDDS Data Server) offering various interfaces to the data that were stored in a data storage system which included backup facilities. This setup allowed DAMOCLES and ACCESS to engage with external projects like the Advanced Cooperative Arctic Data and Information Service (ACADIS^20^) in the US, as well as global programmes like IPYDIS.

The human interface to the data is shown in Fig. S1. It consists of a menu bar on the left side with the search results in the main column. Search results provided direct links to the datasets.

## EXTERNAL INTEROPERABILITY AND FRAMEWORKS: MOVING TOWARDS DISTRIBUTED DATA MANAGEMENT

The traditional way of handling scientific data management has been to use isolated data management systems. Using this approach, data consumers have to visit a number of data management systems to find the relevant data. In recent years, the “silo approach” has been replaced by a network-oriented approach where formerly isolated silos are connected using interoperability technologies. This approach does not affect the basic structure of data repositories, but adds standardised interfaces that can be used to search for, access and combine data from different data repositories. In its simplest form, unified access to metadata, this was implemented for IPY (Parsons et al. 2011a), but future ambitions are higher:

Also necessary, especially in an era when data can be mixed and combined in unanticipated ways, is software that can keep track of which pieces of data came from whom. Such systems are essential if tenure and promotion committees are ever to give credit – as they should – to candidates’ track-record of data contribution. (Editorial, Nature 461: 145 [10 September 2009)].

In recent years, several distributed data management frameworks have emerged. The World Meteorological Organisation (WMO) has developed its WMO Information System,^21^ while the Group on Earth Observations has developed the Global Earth Observations System of Systems^22^ (GEOSS). The Infrastructure for Spatial Information in the European Community^23^ (INSPIRE) is pushing access to public geospatial information in Europe, while discipline-specific data management systems like Global Biodiversity Information Facility^24^ (GBIF) and SeaDataNet^25^ for oceanography have emerged. Some of these frameworks focus primarily on discovery metadata and less on standardisation of use metadata, data encoding and interfaces to data. For distributed data management to fully work, the latter is equally important as the former, although the former has to be solved to make the latter possible.

**Fig. 2:**
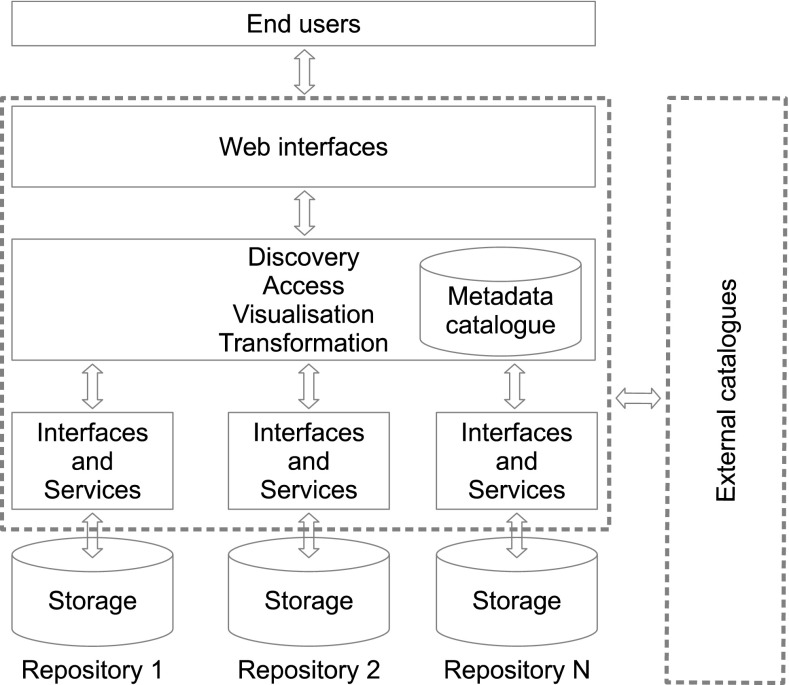
Distributed data management is relying on standardised documentation and interfaces to metadata and data served from the data repositories. This standardisation is bridging between the heterogeneous repositories which is each has its own storage for data. In order to make the system work, interfaces are implemented as services through which the content may be queried or requested

The objective of distributed data management is to link a number of separate data management systems into a virtual data management system that appears as a physical unit to the user community (Fig. 2). What makes the virtual system work is standardisation of documentation (discovery and use metadata), utilisation of controlled vocabularies forcing a common understanding of the concepts used as well as the relation between concepts, and finally standardised interfaces to the information. Another prerequisite for distributed data management is that data are documented and encoded in a standardised manner. This opens for replacing human efforts to find and understand data, in order to combine them into new datasets, using computers—directing the human effort to specifying the rules to use in order to find data and to combine data (including specifying which data to skip for various reasons). There will never be only one way of documenting data. There will be discipline-specific requirements, framework-specific requirements, etc., but it is important to limit the number of standards in order to enable brokering whether at the semantic or structural level. A major challenge in many of the emerging distributed data management frameworks (e.g., WMO Information System and INSPIRE) is the lack of well-defined controlled vocabularies. Instead of reusing and expanding vocabularies it is observed that new vocabularies emerge and there is little focus on cross-walks between vocabularies, both within and between disciplines. Quite often data are annotated using free text. This is counter to what science continually strives to achieve which is utilisation of common terminology.

**Fig. 3:**
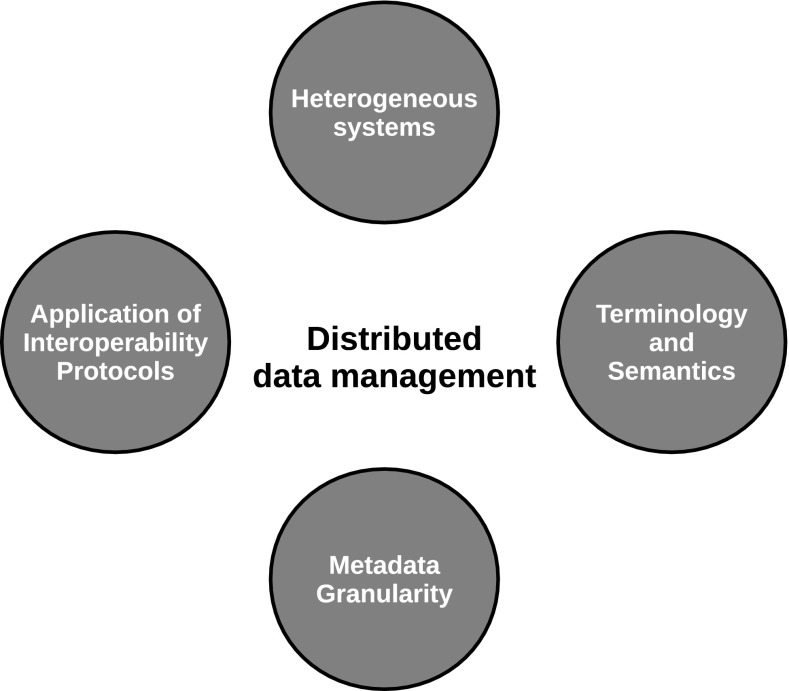
Interoperability emerges in the intersection between science, procedures and technology

While DAMOCLES and ACCESS were not distributed data management systems, the data management system used is capable of participating in distributed data management. Distributed data management is challenged by four issues (Fig. 3). These are heterogeneity, granularity, interoperability and semantics. Data repositories contributing to a distributed data management system are normally heterogeneous. They are often a result of long-term investments by the host institute, investments that cannot easily be redone for the purpose of distributed data management. Furthermore, they have normally only been used by inhouse users or through dedicated user interfaces with access constraints. Frequently this implies that search interfaces are using both discovery and use metadata. While discovery metadata are designed for exchange between data centres, these usually include information on who measured, what, where and when as well as access and use constraints. Within institutional systems, much of this information is implicitly known to users and search interfaces focus more on the content (what and how). When information is integrated across data centres, use metadata is insufficient to guide users in the data discovery process. Furthermore, some data centres provide discovery metadata that are too high level and not useful for data discovery (e.g., you can only search for collections, but not instances within a collection). In order to make useful data discoveries across data repositories, harmonisation of metadata granularity is required. Although it may be useful for a data consumer to know that a data provider has a specific collection of data (e.g., synoptic weather stations), providing direct information and access to the weather stations at Svalbard and that has solar irradiance measurements in the period 1990–2000 is more useful from the perspective of the data consumer. Provision of discovery metadata for each weather station and standardised interfaces to the data allows data management systems to combine data from different providers to a unified dataset. This simplifies the data collection process for scientists leaving more time for the analysis. Finally, within institutional systems, frequently no standardised documentation of the data has been used. Instead, local keywords or identifiers, for example, parameters are used. Without data documentation in a machine-readable form, no automatic translations between standards can be made. Such translations (or brokering) are required to support interdisciplinary science, which is more and more the requirement of funding agencies.

Brokering between data management systems has been tried by, for example, EuroGEOSS (Vaccari et al. 2012; Nativi et al. 2013) and EarthCube/BCube (Khalsa et al. 2013) with more or less success. Often technology development is the driving force instead of the user perspective. INSPIRE, for example, is based on a service-oriented architecture. A service-oriented architecture is based on independent components offering services to each other and communicating through a communication protocol over a network. The problem is that a scientist is not interested in the service, but the data. Instead of looking through a number of services for data, the user should find the relevant datasets and then determine how to access them based on the information provided in the discovery metadata. In other words, the focus should be on content and functionality instead of technology. Furthermore, it is important to remember that scientific data differ in nature. Some datasets are rather static while others describe processes and are thus dynamic. Some processes are slow, while others are very fast. Key scientific issues are evaluated through analysis of time series, spatial collections or other large collections of data. When combining data and analysing them, the relevant data have to be mapped to common data model. The data model is an organisation of data and standardises how they relate to each other. It clearly identifies the characteristics (e.g., variables, units, dimensions) of data and through this simplifies processing and comparison of data. The data model can be very simple and focused on the specific problem and the scientist working on it in a personal analysis tool, or a complex generic model allowing integration between a number of sources. Within the natural sciences, most scientists are trained to think in terms of dimensions and use semantic attributes to describe dimensions and features represented by these dimensions. The Common Data Model of UNIDATA (2017b) is based on this principle. Building data integration/brokering around this data model and the Open-source Project for a Network Data Access Protocol (OPeNDAP^26^), which is extensively used by Earth scientists, is a very cost-efficient mechanism to achieve data interoperability—provided data are well documented (using, e.g., the CF convention^27^) and encoded.

In order to achieve a unified virtual data management system with more or less seamless access to data regardless of the physical location, the following is required to overcome the challenges of heterogeneity, granularity, terminology and semantics and interoperability: (1) discovery-level metadata has to be provided at the highest possible granularity (i.e., station level, not collection of stations); (2) standardised controlled vocabularies available in machine-readable form have to be used to describe the content of data; (3) standardised interfaces are required for accessing data (i.e., no site specific interfaces); (4) data streaming (not file transfer) linked to a generic data model is cost efficient for integration of data from different data centres.

## LESSONS LEARNED

The basic idea of DAMOCLES and ACCESS data management was to involve the scientists in the data management process. This was done through development of supporting tools and documentation. The idea of letting the scientists do the preparation of the data was founded on the fact that they know the data best, and that by letting them participate, the data management process became more cost efficient and of better quality. In a more normal data management process, scientists often deliver data to a data centre where data managers document and prepare data in cooperation with the scientists. By leaving the first and second steps (documentation and quality control) to the scientists, data managers can focus on developing tools, templates, best practise documentation and how to achieve reproducible research (Sandve et al. 2013). A side effect is that scientists are left with datasets that are better documented for their own purpose/analysis as many scientists have experienced trouble, when revisiting a dataset used previously. Structured documentation does not only help with sharing and preserving data, but also with analysing and using the data. However, although scientists understand and share the intention outlined by Sandve et al. (2013), it adds to their workload without clear incentives supporting the additional workload. An in-depth analysis of scientists’ perspective of these issues has not been made, but a combination of lacking external requirements (e.g., enforced by funding agencies) and good examples of the benefits for the scientists (e.g., through discipline specific implementations) is believed to influence the willingness of scientists to publish and share data. As noted by Cutcher-Gershenfeld et al. (2016), the user community is sensitive to how easy it is to use the tools provided for data and software documentation and sharing. Training of scientists will help, but not without being able to identify a clear benefit for the users. Examples of such benefits can be fulfilling requirements imposed by funding agencies or simplification of the work process for scientists. In an increasingly competitive scientific community (in order to get funding), an additional work load has to come with benefits.

In DAMOCLES, the process of scientists documenting and preparing datasets for sharing and preservation worked quite well, although areas that could be improved were identified. In ACCESS this process was less successful, with very few datasets delivered to the data management system. One reason is believed to be the way work packages were specified. In DAMOCLES, datasets were defined as deliverables from the work packages, and tracking of the flow of datasets among work packages was used as a management mechanism. This approach was not used in ACCESS, leaving it difficult to track datasets and their flow. Thus it is recommended to define datasets as deliverables as this can be a good “Key Performance Indicator” for projects but, more importantly, it also increases the willingness to share data. It was also evident that many scientists did not see the point of sharing data although no one would voice this in plenary sessions, only in personal conversations. Scientists know their own data and in many situations cannot understand that these data are of relevance to anybody else. As no in-depth study has been made on this issue, no clear statement on reasons can be made, but it is believed that this is part of a cultural shift that requires extensive outreach and training activities focusing on the benefits for scientists.

The competence in working with digital data is sometimes insufficient, although tools like R, Matlab and Python with contributed packages for data analysis make this easier. Another issue complicating data sharing is the current business model within science. Scientists are evaluated by publications. The time spent from data collection/production to a published paper may be considerable. In this period, scientists have no incentive to publish their data. They fear that other scientists may “steal” their idea and publish an analysis earlier. However, this is an ethical violation and should be handled as such by the scientific community. Recent efforts to establish data publishing journals (e.g., Scientific Data,^28^ Earth System Science Data^29^ and Polar Data Journal^30^) are helpful as they provide citations for the data provider, but these publications need to be acknowledged by funding agencies as well to gain momentum. Furthermore, it is still most common to first publish a “normal paper” and when this is available, then the “data paper” is released. This is delaying the data sharing and the scientific production, and is also rendering the data of little use in near real-time applications. It is however interesting to note that while data publication was quite normal earlier (papers had data listed in tables), it somewhat disappeared in the 90s and early twenty-first century. Now this is emerging again, but enforced by the scientific journals^31^ in order to quality assure science. Linking data publication and sharing to journals only is however reducing the benefit of scientific data collection and production through constraints on the use of data and delayed data sharing. It increases the turnaround time of climate monitoring in polar regions for example, where much of the data collection is performed by scientists and not operational agencies. A good example of the benefits of free and open data is provided by WMO Resolution 40.^32^ While scientists need funding to do data collection and funding, in most situations, is related to publications, data journals are still considered a helpful tool. However, it would probably be better to have good mechanisms supporting citation of data (including real-time data) and funding agencies acknowledging this in their evaluation of projects.

Another lesson learned is that scientists often claim their data are too specialised for others to use them, saying that the data cannot be encoded and documented in a standardised manner. They are focusing on the differences between data types and not the similarities. It is of course true that most standardisation efforts are incomplete and that no obvious standard is available for some data, but not using the standards available prevents a general uptake of this approach in the community. In order to improve the impact of standards, it is also important to continuously refine these and develop community profiles, best practises, etc. This development is, after the initial phase, a process that is best undertaken in a stepwise approach with active engagement from the scientific community (Edwards et al. 2007). The technological framework established has to be filled with content and this requires a joint effort of both technicians and scientists. Technicians are needed to develop the necessary tools simplifying the process for scientists engaging in standardisation. Scientists need to define the content and semantics of these systems to improve the scientific process. It is to establish a terminology for proper description of the concepts studied and sharing this terminology (controlled vocabulary) internally within the community/discipline as well as translating to the terminology used by other communities/disciplines. Unfortunately, the situation today is that many scientists think that sharing data is sufficiently addressed by providing a visual representation of the data in the form of images or graphs through a web page. The understanding of the concept of discovery metadata increased during IPY, when the bulk of geoscientists probably first exposed to the concept, but still much effort remains (Parsons et al. 2011a, b). Following IPY again, more scientists probably have an understanding of the discovery metadata than use metadata, because they had to provide discovery metadata in order to comply with the IPY Data Policy—and this compliance was a requirement in order to achieve the IPY endorsement of projects. However, use metadata are more important when analysing data. Machine interpretation (e.g., for automated visualisation) and combination of heterogeneous data is a really useful feature for scientists but is dependent on standardised use metadata and is not commonly known in the scientific community. The result during IPY (Parsons et al. 2011a) was that discovery metadata often were available, but there were no real data linked to the discovery metadata.

Gaps today are related to the interaction between humans and the technological frameworks. Experience is gained, data are shared (although reluctantly in many cases) and data preservation is becoming an issue of increased focus. A cultural shift is being observed, but it may loose momentum and even stop unless actively promoted. There is a need to bridge the gap between different communities in order to avoid a divergence of interoperability interfaces being developed. A divergence will increase development and maintenance costs for a truly interdisciplinary system supporting the interdisciplinary science required by society.

Part of the problem is that scientists are not actively engaging as a community in the development of the distributed data management systems. This development is often left to technicians that never use the systems, nor understand the data handled or the purpose of the data. Without active engagement of scientists (data providers and consumers) in the development of data management systems, this will continue to be the situation. Data management without an understanding of the purpose and perspective is a wasted investment. However, in this process, scientists also have to acknowledge that technology and society evolve. Putting data in isolated silos is no longer sufficient, data have to be made available online and in a form that allows combination with other data. This will increase the footprint of scientific activity and help justify the huge public investments in data collection and production. An investment that today is underutilised.

More and more often these days, a research project’s success is measured not just by the publications it produces, but also by the data it makes available to the wider community. Pioneering archives such as GenBank have demonstrated just how powerful such legacy data sets can be for generating new discoveries – especially when data are combined from many laboratories and analysed in ways that the original researchers could not have anticipated. (Editorial, Nature 461:145 [10. September 2009]).

## SUMMARY

The lessons learned in development and operation of a data management system for EU projects DAMOCLES and ACCESS are:

1. Make sure that the most important functionality of the data management system is available before the project starts.
2. Create tools that are easy to use for the data providers.
3. Show the benefit of documentation and standardisation for scientists while they are collecting and preparing data.
4. Make partners committed to publish and share data in standardised form.
5. Use the data management system for project management and review.
6. Make datasets deliverables in projects.
7. Involve scientists (as data providers and consumers) in development and operation of data management systems.
8. Make datasets citable and make funding agencies acknowledge citations of datasets.

But the most important lesson learned is that it is very hard to encourage or make scientists share data. In order to fill the technology with content (to develop controlled vocabularies), the active engagement of the scientific community is required. As mentioned above, outreach and training may help, but not without being able to clearly identify benefits for the scientists. Benefits can be external, e.g., through fulfilling requirements from funding agencies and journals, but even more important internal through simplifying the work flow for scientists through development of tools that meet the requirements of scientists. Rather than being specialised tools, this should focus on simplified integration with relevant toolboxes like Matlab, Python and R (examples from geoscience).

## Electronic supplementary material

Below is the link to the electronic supplementary material.

Supplementary material 1 (PDF 156 kb)

## Footnotes

1
http://www.damocles-eu.org/.

2
http://arcticdata.met.no/.

3
http://classic.ipy.org/Subcommittees/final_ipy_data_policy.pdf.

4
https://www.openaire.eu/opendatapilot.

5
http://inspire.ec.europa.eu/.

6
http://arcticdc.org/.

7
http://www.wmo.int/pages/prog/www/WIS/.

8
http://www.earthobservations.org/geoss.php.

9
http://doi.org/10.5065/D6N014KG.

10
http://www.unidata.ucar.edu/software/netcdf/).

11
http://www.unidata.ucar.edu/software/netcdf/conventions.html.

12
http://cfconvention.org/.

13
http://www.unidata.ucar.edu/software/thredds/current/netcdf-java/CDM/.

14
http://gcmd.nasa.gov/add/difguide/.

15
https://www.openarchives.org/OAI/openarchivesprotocol.html.

16 Global attributes is a mechanism for integration of discovery metadata with the data in NetCDF.

17
http://wiki.esipfed.org/index.php/Attribute_Convention_for_Data_Discovery.

18
http://wiki.esipfed.org/index.php?title=Category:Attribute_Conventions_Dataset_Discovery.

19
http://www.unidata.ucar.edu/software/thredds/current/tds/.

20 March 28 2016, all metadata and data in the ACADIS system were transferred into the new NSF Arctic Data Center system.

21
http://www.wmo.int/pages/prog/www/WIS/.

22
http://www.earthobservations.org/geoss.php.

23
http://inspire.ec.europa.eu/.

24
http://www.gbif.org/.

25
https://www.seadatanet.org/.

26
http://opendap.org/.

27 Suitable for meteorological and oceanographic data, but not covering all aspects yet.

28
http://www.nature.com/sdata/.

29
http://www.earth-system-science-data.net/.

30
https://pdr.repo.nii.ac.jp/.

31 See, e.g., http://guides.library.vu.edu.au/content.php?pid=489543&sid=4015042 (accessed 8 August 2016).

32
http://www.wmo.int/pages/prog/www/ois/Operational_Information/Publications/Congress/Cg_XII/res40_en.html.
