# Supplementary material for: ACCESS climate data management
Source: Ambio. 2017 Oct 24;46(Suppl 3):464–74. doi: 10.1007/s13280-017-0963-1 (PMC5673879; doi:10.1007/s13280-017-0963-1)
Supplement: Supplementary file 1 — Supplementary material 1 (PDF 156 kb) [file 13280_2017_963_MOESM1_ESM.pdf]

Ambio

Electronic Supplementary Material
This supplementary material has not been peer reviewed.

Title: **ACCESS Climate Data Management**

Authors: Øystein Godøy, Bard Saadatnejad

Table S1: Global attributes used in the project DAMOCLES in order to generate Discovery metadata for the human search interface.

Name	Purpose
title	A short description of the data set
abstract	A short summary of the data collection activity and data set. This element may alternatively be provided as the global attribute "comment" in a netCDF file.
topiccategory	A blank separated list of topic keywords describing the dataset. See below for applicable keywords.
keywords	A blank separated list of keywords describing the dataset. See below for list of applicable keywords.
gcmd_keywords	Newline separated list of GCMD scientific keywords describing the various variables. This will be used to categorize the datasets according to the "Topics and variables" menu selection in the metadata search facility. If proper standard names have been used for the variables, data will be mapped for search under Topics and variables even without the gcmd_keywords attribute. For more information see below.
activity_type	Comma separated list of activity types. See list below for applicable descriptions.
Conventions	The metadata convention used, should be "CF-1.0"
product_name	A product name of the dataset.
history	Modification history of the dataset. Should be of the form:<br>2007-05-12 creation<br>2007-06-10 revision and separated by newlines.
area	Area name describing the geographical area being studied. If several area names are used, separate them using comma. See below
southernmost_latitude	Elements to describe a geographical bounding box for the data. Should be a floating point value (decimal degrees).
northernmost_latitude	Elements to describe a geographical bounding box for the data. Should be a floating point value (decimal degrees).
westernmost_longitude	Elements to describe a geographical bounding box for the data. Should be a floating point value (decimal degrees).
easternmost_longitude	Elements to describe a geographical bounding box for the data. Should be a floating point value (decimal degrees).
start_date	Start date and time of the dataset in the form "2007-06-12 12:30:00 UTC"
stop_date	Stop date and time of the dataset in the form "2007-06-12 12:30:00 UTC"
institution	Name of the institution responsible for the dataset. Please use one of the standardised names below (short or long name).
PI_name	Name of the person responsible for the data set.
contact	email address to responsible user support or principal investigator. If the email address of the principal investigator is used, the variable "PI_name" should be set accordingly.
distribution_statement	A distribution statement, see below for a applicable list.
project_name	Name of the project within which the data were collected.

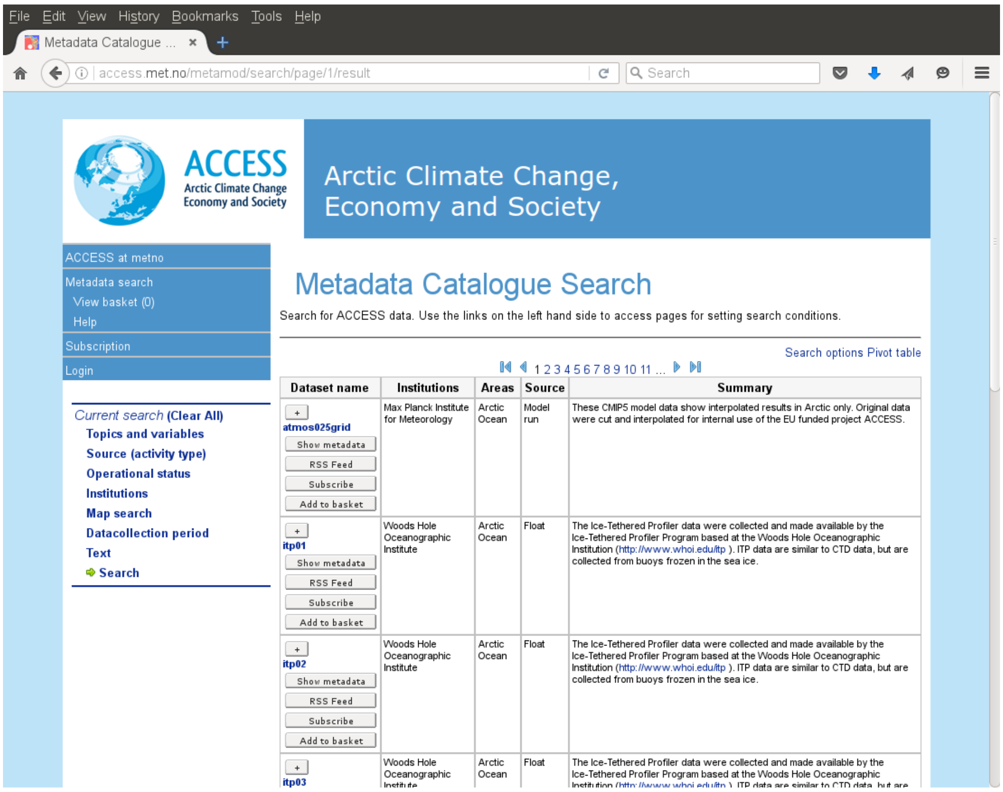

Figure S1: The human search interface developed within DAMOCLES and reused and further developed within ACCESS.
